# Associations of handgrip strength with morbidity and all-cause mortality of cardiometabolic multimorbidity

**DOI:** 10.1186/s12916-022-02389-y

**Published:** 2022-06-03

**Authors:** Yanqiang Lu, Guochen Li, Pietro Ferrari, Heinz Freisling, Yanan Qiao, Luying Wu, Liping Shao, Chaofu Ke

**Affiliations:** 1 Department of Epidemiology and Biostatistics, School of Public Health, Medical College of Soochow University, 199 Renai Road, Suzhou, 215123 People’s Republic of China; 2 Nutrition and Metabolism Branch, International Agency for Research On Cancer (IARC/WHO), Lyon, France

**Keywords:** Handgrip strength, Cardiometabolic multimorbidity, All-cause mortality, UK Biobank

## Abstract

**Background:**

Cardiometabolic multimorbidity (CM) is an increasing public health and clinical concern. However, predictors for the development and prognosis of CM are poorly understood. The aims of this study were to investigate the relation between handgrip strength (HGS) and the risk of CM and to examine the association of HGS with all-cause mortality risk among patients with CM.

**Methods:**

This prospective cohort study involved 493,774 participants from the UK Biobank. CM was defined as the simultaneous occurrence of two or more of the following conditions: type 2 diabetes, stroke, and coronary heart disease (CHD). Cox proportional hazards models were performed to estimate hazard ratios (HRs) and 95% confidence intervals (95% CIs).

**Results:**

During a median follow-up of 12.1 years, 4701 incident CM cases were documented among participants with none cardiometabolic disease at baseline. Compared with the fourth quartile (Q4), the multivariable adjusted HR (95% CI) value of Q1 of HGS for developing CM was 1.46 (1.34–1.60). In participants with one cardiometabolic disease at baseline, participants in Q1 of HGS also possessed higher risk of CM than those in Q4, with HRs (95% CIs) being 1.35 (1.23–1.49) in patients with type 2 diabetes, 1.23 (1.04–1.46) in patients with stroke, and 1.23 (1.11–1.36) in patients with CHD. For participants with CM at recruitment, HGS was also associated with the risk of all-cause mortality (Q1 vs. Q4 HR: 1.57, 95% CI: 1.36–1.80).

**Conclusions:**

Our study provided novel evidence that HGS could be an independent predictor of morbidity and all-cause mortality of CM.

**Supplementary Information:**

The online version contains supplementary material available at 10.1186/s12916-022-02389-y.

## Background

Multimorbidity, defined as the coexistence of at least two chronic diseases in an individual, is an increasing global public health concern mainly due to the aging population trend and urbanization [1–3]. Multimorbidity causes huge burdens for individuals and society, as evidenced by increased rates of disability and mortality, serious psychological problems and high medical expenditures [4–6]. Cardiometabolic multimorbidity (CM) is the most common and harmful pattern of multimorbidity, which refers to simultaneously suffering from more than one of coronary heart disease (CHD), stroke and type 2 diabetes [4, 7]. It is reported that the medical history of CM was associated with a reduction in life expectancy of 15 years at the age of 60, which was almost twice as much as that for any single condition [4]. Furthermore, participants with only one cardiometabolic disease and CM had a 1.4 and 1.9 times higher risk of mental stress than those without cardiometabolic disease, respectively [8]. In spite of the increasing disease burden and alarming health damage, existing studies mainly focus on individual cardiometabolic diseases, and factors predicting CM are poorly understood.

Handgrip strength (HGS), an expedient and low-budget anthropological indicator, is conventionally considered to be an indicator of muscle strength and related to various adverse health events. Many studies have reported that HGS declines from midlife [9, 10] and is highly correlated with new-onset cardiometabolic diseases and early mortality [11–14]. For example, the Prospective Urban Rural Epidemiology (PURE) study holds that HGS was superior to systolic blood pressure in predicting cardiovascular diseases during a four-year follow-up [11]. A study of middle-aged and elderly people in the USA and China reported that for every 0.05 decrease in the normalized grip strength (defined as grip strength divided by weight), the risk of type 2 diabetes increased by 0.49 times [12]. Evidence from the Tromsø Study (*n* = 6850; adults aged 50–80 years and followed up for 17 years) indicated that weaker HGS was associated with increased risks of all-cause mortality and cardiovascular mortality [10]. Previous studies have also assessed the relationship between HGS and cardiometabolic diseases in the UK Biobank [15–17]. Jirapitcha et al. provided evidence that low HGS was associated with a higher risk of incident type 2 diabetes in both men and women [15]. Claire et al. found that HGS and usual walking pace had an additive effect to improve cardiovascular risk prediction [16]. The analysis from a genetic risk perspective also demonstrated that the inverse correlation between HGS and CHD existed in different levels of genetic risk [17]. Therefore, we speculate that HGS may be also an independent predictor for both new-onset CM cases and all-cause mortality events among patients with CM. However, few studies investigated the role of HGS in the whole progression to CM. Moreover, considering the huge reductions in life expectancy among patients with CM, estimating the association of HGS with all-cause mortality in patients with CM would be instructive for implementing tertiary prevention.

To address these limitations, we performed a prospective analysis of population-scale data from the UK Biobank. The objectives of this study are twofold: (1) to examine the association of HGS with the risk of CM in participants with none or only one cardiometabolic disease at recruitment and (2) to investigate the association of HGS with all-cause mortality risk in patients with CM at recruitment. In addition, as sex and age are the most common and important demographic characteristics in epidemiological studies and levels of handgrip strength differ greatly among different sexes and age levels [18, 19], subgroup analyses by sex and age groups were performed to evaluate the robustness of the associations. These subgroup analyses by sex and age groups were specified a priori with the expectation of consistent findings.

## Methods

### Study design and population

Between March 2006 and July 2010, the UK Biobank recruited > 500,000 community-dwelling individuals, aged 37–73 years, from the general population [20, 21]. This prospectively nationwide population-based study collected extensive baseline information via questionnaires, physical measurements, and biological sample testing from 22 dedicated assessment centers across the UK [22, 23]. Details of the UK Biobank have been reported in previous studies [20, 23]. The UK Biobank study was approved by the North West Multi-Centre Research Ethics Committee (REC reference: 11/NW/03820), and all participants gave written informed consent before joining this study.

**Fig. 1:**
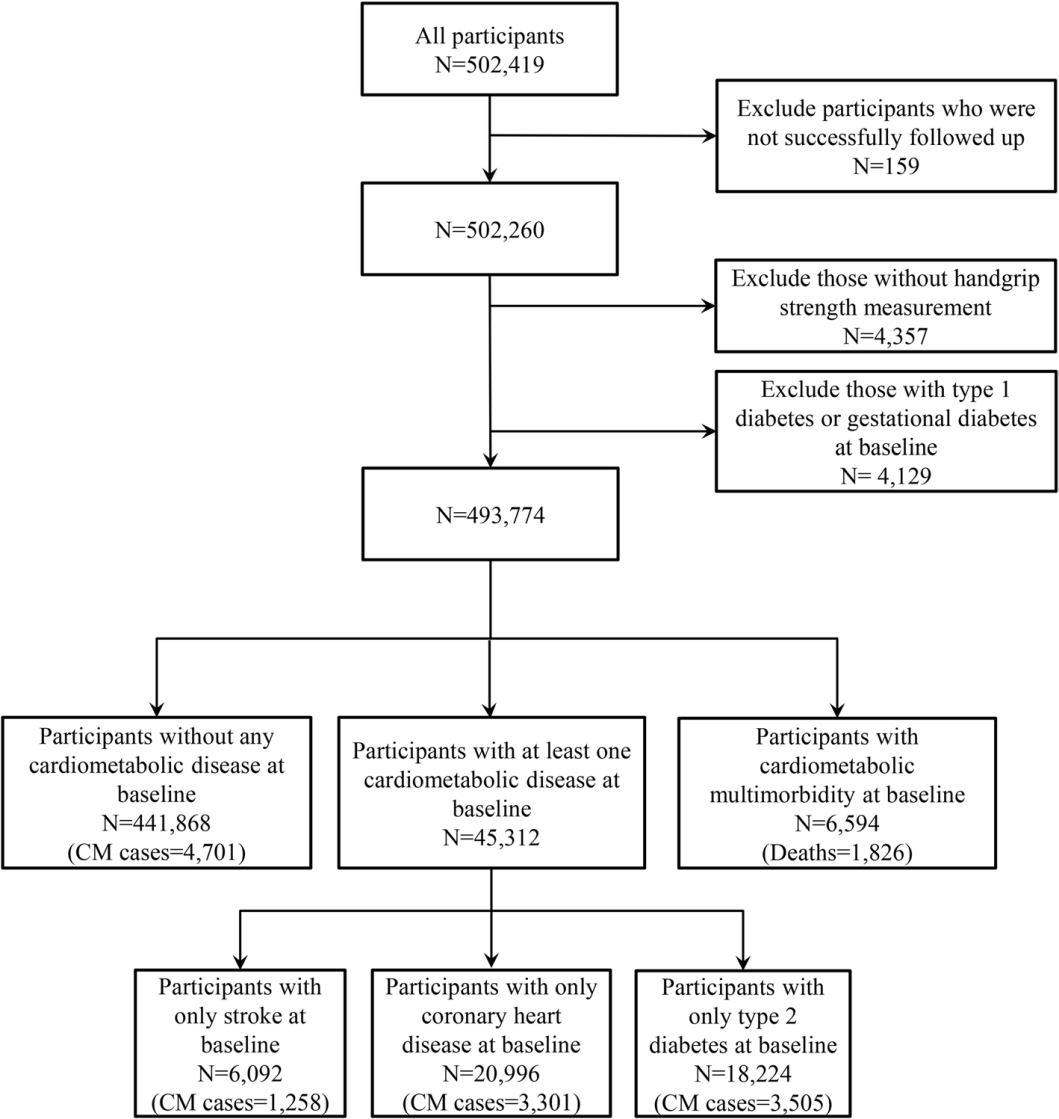
The flowchart of participant selection

In the current study, participants who met any of the following criteria at baseline were excluded: (1) withdrawn from the survey, (2) suffered from type 1 diabetes or gestational diabetes at baseline, and (3) had missing data on HGS. Finally, 441,868 individuals having no cardiometabolic disease, 45,312 individuals with 1 cardiometabolic disease, and 6594 individuals with at least 2 cardiometabolic diseases at baseline were included (Fig. 1).

### Measurement of HGS

HGS was assessed using the Jamar J00105 hydraulic hand dynamometer, with each of the dynamometer calibrated before measurement. Participants were seated upright with their elbows at 90° angles so that their forearms were facing forwards and placed on the armrest. Both left and right handgrip strengths were accurately measured through squeezing the dynamometer as strongly as possible for about 3 s. Participants were also asked about their handedness, and the score from their dominant hand was used in analyses. When the participants failed to indicate a dominant hand, the highest score across both hands was selected and used in analyses. Then, we used the combined quartiles (synthesizing quartile standards for men and women) for analyses on account of the different HGS levels between the two sexes (Additional file 1: Table S1) [24].

### Follow-up of cardiometabolic diseases, CM and mortality

Cardiometabolic diseases were identified from self-reported medical conditions, primary care, linked inpatient hospital data, and death registry records using the tenth edition of the International Classification of Disease (ICD-10) from “first occurrence fields (Category ID: 1712)” as the criteria for the entire cohort from England, Scotland, Wales, and the national health registrations. Specific information can be found online (https://biobank.ndph.ox.ac.uk/showcase/label.cgi?id=1712). In this study, cardiometabolic diseases included type 2 diabetes (E11), stroke (I60-64, I69), and CHD (I20-25), and detailed disease definitions can be seen in Additional file 1: Table S2. The time of CM onset was determined as the date when the second cardiometabolic disease was diagnosed. Date of death was received from death certificates held by the National Health Service (NHS) for participants from England and Wales, and the NHS Central Register (NHSCR), Scotland, for participants from Scotland. At the time of analysis, mortality data were available to 23 March 2021. Further detailed information for the outcomes on the UK Biobank can be found at http://www.ukbiobank.ac.uk. The primary outcomes were CM (at least two from type 2 diabetes, CHD, and stroke) and all-cause mortality.

### Assessment of covariates

Covariates included sociodemographic characteristics and lifestyle factors. Sociodemographic covariates included age (continuous), sex (men and women), ethnicity (classified as White, mixed, Asian/Asian British, Black/Black British, and others), and the Townsend deprivation index. The Townsend deprivation index, derived from the postcode of residence, was used to describe the area-based socioeconomic status through the quartiles of indices [25]. Lifestyle covariates were measured by self-report, including smoking status (classified as never, former and current smoking) and frequency of alcohol intake (divided into never, special occasions only, one to three times a month, once or twice a week, three or four times a week, and daily). Physical activity was assessed with the International Physical Activity Questionnaire (IPAQ) short form [26]. The volume of physical activity was calculated as the sum of walking, moderate and vigorous activity over the previous week, measured as metabolic equivalents (METs min/week) [27]. A MET-minute is computed by multiplying the MET score of an activity by the minutes performed. As recommended by IPAQ guidelines, the MET scores were 3.3 for walking, 4.0 for moderate physical activity, and 8.0 for vigorous physical activity. Physical activity was categorized into three groups: low (MET < 600 min/week), moderate (600 to 3000 MET), and high (MET > 3000) [26]. Hypertension was defined using the combination of the self-reported history of antihypertensive medication use, medical diagnosis records, and clinical measurements of blood pressure (systolic blood pressure (SBP) ≥ 130 mmHg or diastolic blood pressure (DBP) ≥ 80 mmHg). Furthermore, height and body weight were measured by well-trained nurses during the initial assessment center visit. We calculated body mass index (BMI) as weight (kg) divided by the square of height (m) and divided BMI into four categories according to the recommendations of the World Health Organization (WHO): underweight (< 18.5 kg/m^2^), normal weight (18.5 to < 25), overweight (25 to < 30), and obesity (≥ 30). Further details of these measurements can be found in the online UK Biobank Showcase (http://biobank.ndph.ox.ac.uk/showcase).

### Statistical analysis

For participant baseline characteristics, we described continuous data as mean and SD and calculated the frequencies and percentages for categorical variables.

Follow-up time was from the baseline date to the diagnosis date of the outcomes, date of death, or the censoring date (March 23, 2021), whichever came first. Kaplan–Meier curves stratified by HGS quartiles are showed in Additional file 1: Fig. S1. The proportionality of hazards assumption of Cox regression model was tested by Schoenfeld residuals method. Multivariable cubic regression splines were used to evaluate the linearity assumption for the continuous exposure (per 1 SD), and we found no evidence of deviation from linearity.

**Fig. 2:**
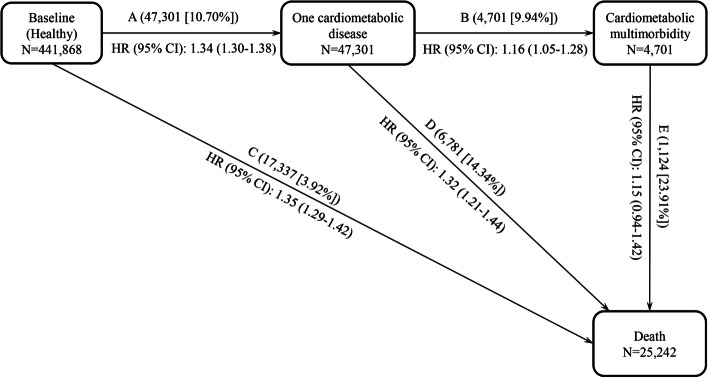
Associations of handgrip strength with risks of all transitions from baseline to individual cardiometabolic disease, then to subsequent CM and ultimately to death. Note: HR values correspond to the hazards ratios of the first quartile of handgrip strength to the fourth quartile. Models were adjusted for age, sex, socioeconomic status, ethnicity, smoking status, alcohol drinking, physical activity, BMI, and hypertension

The associations of HGS (per 1 SD decrease and in quartiles, Q1-Q4) with CM and all-cause mortality were estimated by Cox proportional hazards models. For participants who have none cardiometabolic disease at recruitment (*N* = 441,868), we examined the association of HGS with onset CM. The multistate model was performed to evaluate the associations of baseline HGS with risks of all transitions from a “healthy” state to one cardiometabolic disease, then to subsequent CM and ultimately to all-cause mortality (Fig. 2). And the multistate model included five transitions: (A) from baseline to one cardiometabolic disease, (B) from one cardiometabolic disease to CM, (C) from baseline to death, (D) from one cardiometabolic disease to death, and (E) from CM to death. In subsequent analyses, we also examined the risk of CM associated with HGS among individuals with only one cardiometabolic disease (type 2 diabetes, stroke or CHD, *N* = 45,312) at baseline. Finally, the risk magnitude of HGS for all-cause mortality was estimated among baseline CM participants (*N* = 6594). All results are presented as hazard ratios (HRs) and 95% confidence intervals (CIs).

Multivariable-adjusted models included age, sex, socioeconomic status, ethnicity, smoking status, alcohol drinking, physical activity, BMI, and hypertension. Blood lipids were not included in the adjusted model, since they may be potential mediators that are caused by the exposure and in turn cause the outcome. We used multivariate imputations by chained equations (MICE) based on random forest to fill in the missing values of the covariates [28]. The imputation models used waist circumference, sleep duration, average total household income before tax, forced vital capacity, albumin, and creatinine as the auxiliary variables. We performed 20 iterations and generated 5 datasets to account for the missing data at baseline (Additional file 1: Tables S3 and S4) and combined the estimates of the 5 datasets according to Rubin’s rules [29]. Potential sex, age, or physical activity interactions with HGS were computed by the likelihood ratio test to compare models with and without cross-product interaction terms (Additional file 1: Table S5). Subgroup analyses by age (< 60 *vs.* ≥ 60), sex (men *vs.* women) and physical activity (low *vs.* moderate *vs.* high) were performed to evaluate the robustness of the findings. The subgroup analyses by physical activity levels were post-hoc analyses following peer-review without a pre-specified hypothesis.

Furthermore, we conducted a sensitivity analysis by adding a new variable = entry_time + 2 (years) as the entry time and removing the participants with a follow-up time less than zero to minimize the potential reverse causation and induction time bias.

All analyses were performed using SAS 9.4 (SAS Institute, Cary, NC, USA) and R software (4.0.5).

## Results

### From no cardiometabolic disease at recruitment to CM

A total of 441,868 participants without any cardiometabolic disease at baseline were included in this stage. Over a median follow-up period of 12.1 years, 4701 (1.06%) participants developed CM. The main characteristics of the participants without any cardiometabolic disease at recruitment are summarized in Table 1.

**Table 1:** Baseline characteristics of the study population

	Without any cardiometabolic disease (*N* = 441,868)	With type 2 diabetes alone (*N* = 18,224)	With stroke alone (*N* = 6092)	With CHD alone (*N* = 20,996)	With CM (*N* = 6594)
Male (%)	192,269 (43.51)	10,992 (60.32)	3278 (53.81)	14,068 (67.00)	4758 (72.16)
Age (years)	56.03 (8.10)	59.62 (6.99)	60.12 (7.05)	61.71 (6.18)	62.09 (5.84)
Ethnic (%)					
White	418,046 (94.61)	15,615 (85.68)	5853 (96.08)	19,923 (94.89)	5879 (89.16)
Mixed	2623 (0.59)	128 (0.70)	21 (0.34)	79 (0.38)	39 (0.59)
Asian or Asian British	7361 (1.67)	1197 (6.57)	70 (1.15)	490 (2.33)	378 (5.73)
Black or Black British	6642 (1.50)	700 (3.84)	91 (1.49)	221 (1.05)	136 (2.06)
Other	5199 (1.18)	423 (2.32)	32 (0.53)	176 (0.84)	111 (1.68)
“Do not know” or missing	1997 (0.45)	161 (0.88)	25 (0.41)	107 (0.51)	51 (0.77)
Townsend score	− 1.39 (3.04)	− 0.49 (3.41)	− 0.82 (3.31)	− 0.81 (3.32)	− 0.01 (3.54)
Smoking (%)					
Never smoked	247,314 (55.97)	8598 (47.18)	2689 (44.14)	8283 (39.45)	2215 (33.59)
Previous smoker	146,932 (33.25)	7520 (41.26)	2444 (40.12)	10,015 (47.70)	3376 (51.20)
Current smoker	45,595 (10.32)	1917 (10.52)	915 (15.02)	2530 (12.05)	930 (14.10)
“Do not know” or missing	2027 (0.46)	189 (1.04)	44 (0.72)	168 (0.80)	73 (1.11)
Drinking (%)					
Daily or almost daily	91,273 (20.66)	2637 (14.47)	1237 (20.31)	4282 (20.39)	1007 (15.27)
Three or four times a week	104,627 (23.68)	2802 (15.38)	1118 (18.35)	4461 (21.25)	978 (14.83)
Once or twice a week	115,042 (26.04)	4201 (23.05)	1506 (24.72)	5130 (24.43)	1477 (22.40)
One to three times a month	49,190 (11.13)	2248 (12.34)	676 (11.10)	2101 (10.01)	734 (11.13)
Special occasions only	48,575 (10.99)	3430 (18.82)	817 (13.41)	2695 (12.84)	1171 (17.76)
Never	32,269 (7.30)	2817 (15.46)	720 (11.82)	2268 (10.80)	1199 (18.18)
“Do not know” or missing	892 (0.20)	89 (0.49)	18 (0.30)	59 (0.28)	28 (0.42)
Physical activity (%)					
Low	64,171 (14.52)	3688 (20.24)	1036 (17.01)	3441 (16.39)	1577 (23.92)
Moderate	181,083 (40.98)	6816 (37.40)	2218 (36.41)	7864 (37.45)	2189 (33.20)
High	110,769 (25.07)	3565 (19.56)	1374 (22.55)	5018 (23.90)	1216 (18.44)
“Do not know” or missing	85,845 (19.43)	4155 (22.80)	1464 (24.03)	4673 (22.26)	1612 (24.45)
BMI (kg/m^2^)	27.11 (4.60)	31.53 (5.89)	28.10 (4.77)	28.83 (4.77)	31.34 (5.64)
HGS (Male, kg)	42.22 (8.91)	38.60 (9.25)	38.80 (9.13)	39.27 (8.69)	36.39 (9.19)
HGS (Female, kg)	25.28 (6.35)	22.88 (6.57)	22.82 (6.67)	22.22 (6.39)	20.90 (6.69)
SBP (mmHg)	137.53 (18.66)	141.80 (16.95)	140.01 (18.64)	138.46 (18.69)	139.24 (18.74)
DBP (mmHg)	82.37 (10.11)	82.49 (9.37)	82.59 (10.05)	79.74 (10.22)	79.04 (10.36)
HDL (mmol/L)	1.47 (0.38)	1.20 (0.31)	1.39 (0.37)	1.27 (0.34)	1.14 (0.31)
LDL (mmol/L)	3.65 (0.83)	2.75 (0.78)	3.05 (0.88)	2.90 (0.82)	2.64 (0.74)
Triglycerides (mmol/L)	1.72 (1.01)	2.14 (1.20)	1.73 (0.99)	1.86 (1.03)	2.21 (1.28)

Data were presented as frequency (%) or mean (standard deviation).

*Abbreviations*: *CHD* coronary heart disease, *CM* cardiometabolic multimorbidity, *BMI* body mass index, *HGS* handgrip strength, *HDL* high-density lipoprotein, *LDL* low-density lipoprotein, *SBP* systolic blood pressure, *DBP* diastolic blood pressure

As shown in Table 2, after multivariable adjustment (model 3), per 1 SD lower HGS was positively associated with the risk of CM (HR: 1.17, 95% CI: 1.14–1.21). Compared with the fourth quartile (Q4), the HR (95% CI) values of Q1 of HGS for developing CM were 1.46 (1.34–1.60). The multistate model revealed that HGS (Q1 *vs.* Q4) was associated with risks of all transitions from baseline to individual cardiometabolic disease, then to subsequent CM and ultimately to death, except the transition from CM to all-cause mortality (transition E, Fig. 2 and Additional file 1: Table S6). The multivariable adjusted HR (95% CI) values of HGS were 1.34 (1.30–1.38) for transition A, 1.16 (1.05–1.28) for transition B, 1.35 (1.29–1.42) for transition C, 1.32 (1.21–1.44) for transition D, and 1.15 (0.94–1.42) for transition E, respectively.

**Table 2:** Associations of handgrip strength with the risk of cardiometabolic multimorbidity

	Unadjusted model		Adjusted model	
	HR (95% CI)	*P*	HR (95% CI)	*P*
**Participants without any cardiometabolic disease at baseline (Participants=441,868 / CM cases=4,701)**				
Q4 (highest)	1 (reference)		1 (reference)	
Q3	1.32 (1.21–1.45)	< 0.0001	1.08 (0.99–1.19)	0.0943
Q2	1.70 (1.56–1.86)	< 0.0001	1.23 (1.13–1.34)	< 0.0001
Q1 (lowest)	2.41 (2.22–2.62)	< 0.0001	1.46 (1.34–1.60)	< 0.0001
Continuous variable^a^	1.21 (1.18–1.24)	< 0.0001	1.17 (1.14–1.21)	< 0.0001
**Participants with type 2 diabetes at baseline (Participants=18,224 / CM cases=3,505)**				
Q4 (highest)	1 (reference)		1 (reference)	
Q3	1.06 (0.96–1.17)	0.2774	1.01 (0.91–1.11)	0.8936
Q2	1.38 (1.26–1.51)	< 0.0001	1.15 (1.05–1.26)	0.0039
Q1 (lowest)	1.71 (1.56–1.88)	< 0.0001	1.35 (1.23–1.49)	< 0.0001
Continuous variable	1.14 (1.10–1.17)	< 0.0001	1.13 (1.09–1.17)	< 0.0001
**Participants with stroke at baseline (Participants=6,092 / CM cases=1,258)**				
Q4 (highest)	1 (reference)		1 (reference)	
Q3	1.02 (0.87–1.20)	0.7725	0.97 (0.82–1.13)	0.6687
Q2	1.24 (1.06–1.45)	0.0068	1.06 (0.90–1.24)	0.4766
Q1 (lowest)	1.53 (1.30–1.80)	< 0.0001	1.23 (1.04–1.46)	0.0159
Continuous variable	1.08 (1.02–1.13)	< 0.0001	1.10 (1.03–1.16)	0.0021
**Participants with CHD at baseline (Participants=20,996 / CM cases=3,301)**				
Q4 (highest)	1 (reference)		1 (reference)	
Q3	1.16 (1.05–1.28)	0.0025	1.14 (1.03–1.25)	0.0110
Q2	1.24 (1.13–1.37)	< 0.0001	1.15 (1.05–1.27)	0.0045
Q1 (lowest)	1.42 (1.29–1.57)	< 0.0001	1.23 (1.11–1.36)	< 0.0001
Continuous variable	1.08 (1.04–1.11)	< 0.0001	1.09 (1.05–1.13)	< 0.0001

Adjusted model was adjusted for age, sex, socioeconomic status, ethnicity, smoking status, alcohol drinking, physical activity, BMI and hypertension

^a^Continuous variable was represented by per standard deviation (SD) decrease of handgrip strength

*Abbreviations*: *HR* hazard ratio, *CI* confidence interval, *Q1* the first quartile, *Q2* the second quartile, *Q3* the third quartile, *Q4* the last quartile, *CHD* coronary heart disease, *CM* cardiometabolic multimorbidity

### From one cardiometabolic disease at recruitment to CM

Among 45,312 individuals with one cardiometabolic disease at baseline, 3505 (19.23%) out of 18,224 participants developed CM from type 2 diabetes; 1258 (20.65%) out of 6092 participants had CM from stroke; and 3301 (15.72%) out of 20,996 participants experienced CM from CHD. Baseline characteristics of the study population are presented in Table 1 by different baseline disease conditions. The mean age of the CHD group was older than the other two groups (61.71 years old for CHD *vs.* 60.12 years old for stroke *vs.* 59.62 years old for type 2 diabetes). Likewise, the proportion of men among patients with CHD was the highest (CHD 67.00%; type 2 diabetes 60.32%; stroke 53.81%). However, levels of physical activity, BMI, HDL, SBP, DBP, and triglyceride did not meet this pattern.

For individuals with type 2 diabetes at the time of inclusion, the multivariable adjusted HRs (95% CI) the HGS of Q3-Q1 (Q4 as the reference) were equal to 1.01 (0.91–1.11), 1.15 (1.05–1.26), and 1.35 (1.23–1.49), respectively (Table 2).

Patients with stroke at baseline were then followed up for CM outcomes. Lower levels of HGS were associated with an increased risk of CM and the HR (95% CI) value of Q1 of HGS for CM was 1.23 (1.04–1.46) after full adjustment when compared to Q4 (Table 2).

In the fully adjusted model (model 3), positive associations were observed between low HGS and the risk of new-onset CM in patients with CHD at recruitment. Individuals in Q3-Q1 of HGS had 1.14 (1.03–1.25), 1.15 (1.05–1.27), and 1.23 (1.11–1.36) times the risk of developing CM than those in Q4, respectively (Table 2).

### From CM at recruitment to all-cause mortality

Among 6594 individuals with CM at baseline, the mean age was 62.09 ± 5.84 years, and 1826 (27.69%) died over a median follow-up period of 11.7 (10.8–12.6) years. Of those, men were more likely than women to experience CM, with 72% of men and 28% of women, and the average HGS values were 36.39 ± 9.19 for men and 20.90 ± 6.69 for women (Table 1).

When compared to Q4, the unadjusted HR (95% CI) of Q1 of HGS for all-cause mortality (model 1) was 1.87 (95% CI: 1.64 to 2.14). This association was slightly attenuated, but remained after full adjustment (HR: 1.57, 95% CI: 1.36–1.80; Table 3).

**Table 3:** Association of handgrip strength with the risk of all-cause mortality among 6594 patients with cardiometabolic multimorbidity

	Unadjusted model		Adjusted model	
	HR (95% CI)	*P*	HR (95% CI)	*P*
Q4 (highest)	1 (reference)		1 (reference)	
Q3	1.31 (1.15–1.49)	< 0.0001	1.19 (1.05–1.36)	0.0088
Q2	1.47 (1.28–1.68)	< 0.0001	1.21 (1.05–1.39)	0.0072
Q1 (lowest)	1.87 (1.64–2.14)	< 0.0001	1.57 (1.36–1.80)	< 0.0001
Continuous variable^a^	1.12 (1.08–1.17)	< 0.0001	1.15 (1.10–1.21)	< 0.0001

Adjusted model was adjusted for age, sex, socioeconomic status, ethnicity, smoking status, alcohol drinking, physical activity, BMI, and hypertension

^a^Continuous variable was represented by per standard deviation (SD) decrease of handgrip strength

*Abbreviations*: *HR* hazard ratio, *CI* confidence interval, *Q1* the first quartile, *Q2* the second quartile, *Q3* the third quartile, *Q4* the last quartile

### Subgroup and sensitivity analyses

In the subgroup analyses by age (< 60 *vs.* ≥ 60), sex (men *vs.* women), and physical activity (low *vs.* moderate *vs.* high), low HGS still possessed a high risk of CM among participants with none or only one cardiometabolic disease (Additional file 1: Figs. S2-S7), with several exceptions among patients with stroke (e.g., in the group of age under 60 years). Meanwhile, associations of HGS with all-cause mortality among patients with CM persisted in nearly all sub-groups (Additional file 1: Figs. S8-S10). In addition, we also observed a similar pattern of results in the sensitivity analyses for HGS after adding a new variable = entry_time + 2 (years) as the entry time (Additional file 1: Tables S7 and S8).

## Discussion

We examined the associations of HGS with morbidity and all-cause mortality of CM in a large national sample of UK adults. Our results revealed that lower HGS in individuals with none or a single cardiometabolic disease at baseline was positively associated with the risk of CM. In addition, a lower HGS was positively associated with all-cause mortality in people who experienced CM.

The current study extended previous studies by reporting a novel finding that lower HGS among participants free of any cardiometabolic disease was associated with a higher risk of CM. Many previous studies have found inverse correlations of HGS with risks of cardiovascular disease and type 2 diabetes [18, 30–33], and muscle strengthening exercise for improving physical fitness, which is correlated with HGS, has been considered as a cost-effective prevention strategy for cardiovascular disease and type 2 diabetes [34–36]. Using data from the UK Biobank, Carlos et al. reported a negative dose–response relationship between HGS and the risk of cardiovascular disease, and the HRs (95% CIs) were 1.15 (1.13–1.17) increased for women and 1.11 (1.10–1.12) increased for men per 5 kg lower HGS, respectively [18]. A systematic review and meta-analysis conducted on 177,826 participants demonstrated that the relative risk (RR) of developing type 2 diabetes in the top tertile of HGS was decreased by 27% (16–37%), in comparison with the bottom tertile [37]. In the current study, we observed not only the individual associations of HGS on cardiovascular disease and type 2 diabetes, but also an association of HGS with CM. Given this finding, screening and monitoring for CM may be instructive among “healthy” people with low HGS.

Our finding of the inverse correlation between HGS and new-onset CM in participants with preexisting type 2 diabetes was also in agreement with previous research [31, 38, 39]. Carlos et al. reported that compared with the highest tertile of HGS in patients with type 2 diabetes, the HR (95% CI) value of the lowest tertile for new-onset cardiovascular disease was 1.87 (1.43–2.46) [38]. Similarly, a retrospective clinical cohort study in Japan also demonstrated that HGS had an inverse association with the occurrence of cardiovascular disease in patients with type 2 diabetes at baseline [31]. However, when the sex-stratified analysis in this study was performed, the association disappeared in both men and women, probably due to the relatively small sample sizes or short follow-up periods. Our prospective analyses of the UK Biobank also provide evidence for the association of HGS with new-onset cardiovascular disease in patients with diabetes.

Remarkably, as far as our information goes, the associations of HGS with the process of developing new-onset type 2 diabetes in patients with stroke or CHD has not been validated. However, the Emerging Risk Factors Collaboration indicated when compared to healthy individuals that the HR for all-cause mortality was about twice in participants with either stroke or myocardial infarction, whereas the risk was almost 4 times in participants with any two of stroke, type 2 diabetes and myocardial infarction [4]. Therefore, our findings suggest that HGS could be an effective and practical indicator to monitor for CM not only in patients with type 2 diabetes but also in patients with stroke or CHD. Screening and interfering in patients who have low HGS with one cardiometabolic disease could be also important to prevent the development of CM.

To our knowledge, prior studies mainly focused on the predictive value of HGS on all-cause mortality among participants with none or one single cardiometabolic disease [11, 32, 38, 40, 41]. Nevertheless, previous studies have already demonstrated that individuals with CM shared a shorter life expectancy than those with a single cardiometabolic disease [4, 42]. Giving that HGS was demonstrated to be an independent predictor of all-cause mortality among patients with CM in our study, interfering in patients with low HGS who already experienced CM might be useful to prevent subsequent mortality.

The underlying mechanisms for the associations of HGS with CM have not been fully elucidated but may be explained from several aspects. First, low HGS is closely related to an early onset of obesity, and obesity increases the risk of dyslipidemia and systemic inflammation, which may be a common route for the development of diabetes, vascular diseases, and their serious complications [43, 44]. Second, HGS is closely linked with undesirable cardiometabolic markers, such as glycosylated hemoglobin (HbA1c) and uric acid (UA) [45, 46]. In addition to facilitating the diagnosis of diabetes, HbA1c is also used to explain the occurrence of CHD and ischemic stroke in many areas [47]. Many epidemiological studies also showed that there was an association between elevated serum UA levels and cardiovascular disease. Intermediate processes may include the production of excessive UA, leading to increased oxidative stress, vasoconstriction, and vascular smooth muscle cell proliferation [48, 49]. Third, a variety of myokines secreted during skeletal muscle exercise also have the effect of regulating the body’s metabolism. For example, functional deficiency of IL-6 may lead to atherosclerotic lipid conditions and insulin resistance [50, 51].

Benefiting from the large sample size and long follow-up periods of the UK Biobank, we were able to investigate the role of baseline HGS in the whole progression to CM more thoroughly. Our study is the first to survey the relationship between baseline HGS and future CM among participants free of cardiometabolic diseases or those with stroke or CHD at recruitment. In addition, this study adds new knowledge that HGS could serve as an independent predictor of all-cause mortality among patients with CM at recruitment. However, we must be aware of some limitations in our study as well. First, as a population-based cohort study, the UK Biobank is not fully representative of the general population. However, a recent study has found the risk factor associations derived from the UK Biobank are basically in line with other representative cohorts [52]. Second, although several common potential confounders associated with cardiovascular disease and diabetes had been adjusted at baseline, there might be other confounding factors that would affect the outcomes. Third, the survival bias may exist in this study since individuals with lower HGS are more likely to have died before the recruitment [11, 18, 53], and the failure to include these individuals could result in the underestimation for the associations. Fourth, the HGS measurement may be more affected in patients with stroke as compared to others, as long-term disability is the most frequent complication after stroke [54]. Fifth, subgroup analyses stratified by physical activity levels were post-hoc analyses which are less credible than pre-specified subgroup analyses [55]. Sixth, the reverse association of HGS with the risk of CM from individuals with stroke at baseline was observed in the group of age < 60. The current findings also warrant further validation in patients with stroke with larger sample sizes. Last, although some studies suggested that using this approach of defining outcomes by ICD-10 codes might slightly underestimate the cases of common diseases, it has the advantage of being more in line with the actual health care system [56, 57].

## Conclusions

In conclusion, this current study revealed an inverse relationship between HGS and new-onset CM in a large population-based cohort. Moreover, low HGS was also associated with a high risk of all-cause mortality among patients with CM. In view of the severe consequences of CM, our study may have far-reaching significance in public health and clinical settings. The findings highlight that HGS may be a modifiable predictive factor in the development and progression of CM. Future studies are needed to validate whether it is instructive to screen high-risk individuals through HGS and conduct indicated intervention to improve physical fitness in healthy people, in people with a cardiometabolic condition and in people with CM.

## Supplementary Information

**Additional file 1:** 

## Data Availability

The data that support the findings of this study are available from the UK Biobank (https://www.ukbiobank.ac.uk/), subject to successful registration and application process. This study was conducted using the UK Biobank resource under application number 60651.

## Abbreviations

BMI: Body mass index
CHD: Coronary heart disease
CI: Confidence interval
CM: Cardiometabolic multimorbidity
DBP: Diastolic blood pressure
HbA1c: Glycosylated hemoglobin
HDL: High-density lipoprotein
HGS: Handgrip strength
HR: Hazard ratio
MET: Metabolic Equivalent Task
SBP: Systolic blood pressure
SD: Standard deviation
Q1: The first quartile
Q2: The second quartile
Q3: The third quartile
Q4: The fourth quartile
UA: Uric acid
