# Supplementary material for: Associations of handgrip strength with morbidity and all-cause mortality of cardiometabolic multimorbidity
Source: BMC Med. 2022 Jun 3;20:191. doi: 10.1186/s12916-022-02389-y (PMC9164350; doi:10.1186/s12916-022-02389-y)
Supplement: Supplementary file 1 — Additional file 1: [file 12916_2022_2389_MOESM1_ESM.docx]

**Additional file 1**

**Associations of handgrip strength** **with** **morbidity and all-cause mortality of cardiometabolic multimorbidity**

Yanqiang Lu^1#^, Guochen Li^1#^, Pietro Ferrari^2^, Heinz Freisling^2^, Yanan Qiao^1^, Luying Wu^1^, Liping Shao^1^, and Chaofu Ke^1^^*^

*^1^* *Department of* *Epidemiology and Biostatistics, School of Public Health, Medical College of Soochow University, 199 Renai Road, Suzhou 215123, P. R. China.*

*^2^ Nutrition and Metabolism Branch, International Agency for Research on Cancer (IARC/WHO), Lyon, France*

^#^ Yanqiang Lu and Guochen Li made equal contributions to this work.

*Correspondence should be addressed to:

Chaofu Ke, PhD

Tel.: +86-512-6588-0079

E-mail: cfke@suda.edu.cn

Department of Epidemiology and Biostatistics, School of Public Health, Medical College of Soochow University, 199 Renai Road, Suzhou 215123, P. R. China.

**Table S1** Cut-off points for sex-specific handgrip strength quartiles

| Sex | Type of baseline disease | Q1 (Lowest) | Q2 | Q3 | Q4 (Highest) |
| --- | --- | --- | --- | --- | --- |
| Women | Participants without  cardiometabolic disease | <21 | 21-26 | 26-30 | ≥30 |
|  | Participants with type 2 diabetes | <18 | 18-22 | 22-28 | ≥28 |
|  | Participants with stroke | <18 | 18-22 | 22-28 | ≥28 |
|  | Participants with CHD | <18 | 18-22 | 22-26 | ≥26 |
|  | Participants with CM | <16 | 16-20 | 20-26 | ≥26 |
| Men | Participants without  cardiometabolic disease | <36 | 36-42 | 42-48 | ≥48 |
|  | Participants with type 2 diabetes | <32 | 32-39 | 39-44 | ≥44 |
|  | Participants with stroke | <32 | 32-39 | 39-45 | ≥45 |
|  | Participants with CHD | <34 | 34-40 | 40-45 | ≥45 |
|  | Participants with CM | <30 | 30-36 | 36-42 | ≥42 |

Note: CHD, coronary heart disease; CM, cardiometabolic multimorbidity; Q1, the first quartile; Q2, the second quartile; Q3, the third quartile; Q4, the last quartile.

**Table S2** Definitions of the three cardiometabolic diseases

|  | **Baseline** | **Incident** |
| --- | --- | --- |
| Type 2 diabetes* | 2443, 2976, 2986, 4041, 6153, 6177, 20002, 20003  ICD-10: E10, E11, E12, E13, E14, O24 | ICD-10: E11 |
| Coronary heart disease | 6150, 20002  ICD-10: I20, I21, I22, I23, I24, I25 | ICD-10: I20, I21, I22, I23, I24, I25 |
| Stroke | 6150, 20002  ICD-10: I60, I61, I62, I63, I64, I69 | ICD-10: I60, I61, I62, I63, I64, I69 |

*Baseline type 2 diabetes was assessed by the diabetes diagnosis algorithms in the UK Biobank (Algorithms for the Capture and Adjudication of Prevalent and Incident Diabetes in UK Biobank. DOI: 10.1371/journal.pone.0162388).

**Table S3** Description of the missing data

|  | Missing values |  | Percentage of missingness (%) |
| --- | --- | --- | --- |
| Sex (%) | 0 |  | 0 |
| Age (years) | 0 |  | 0 |
| Ethnic (%) | 2,341 |  | 0.47 |
| Townsend score | 609 |  | 0.12 |
| Smoking (%) | 2,501 |  | 0.51 |
| Drinking (%) | 1,086 |  | 0.22 |
| Physical activity (%) | 97,749 |  | 19.80 |
| BMI (kg/m^2^) | 1,153 |  | 0.23 |
| SBP (mmHg) | 43,940 |  | 8.90 |
| DBP (mmHg) | 43,928 |  | 8.90 |

Abbreviations: BMI, body mass index; SBP, systolic blood pressure; DBP, diastolic blood pressure.

**Table S4** Baseline characteristics of the study population before and after imputation

|  | **Before imputation** | **After imputation** (the first dataset) | **After imputation**  (the second dataset) | **After imputation** (the third dataset) | **After imputation** (the fourth dataset) | **After imputation** (the fifth dataset) |
| --- | --- | --- | --- | --- | --- | --- |
| Male (%) | 225,365 (45.64) | 225,365 (45.64) | 225,365 (45.64) | 225,365 (45.64) | 225,365 (45.64) | 225,365 (45.64) |
| Age (years) | 56.53 (8.09) | 56.53 (8.09) | 56.53 (8.09) | 56.53 (8.09) | 56.53 (8.09) | 56.53 (8.09) |
| Ethnic (%) |  |  |  |  |  |  |
| White | 465,316 (94.24) | 467,501 (94.68) | 467,497 (94.68) | 467,499 (94.68) | 467,487 (94.68) | 467,474 (94.67) |
| Mixed | 2,890 (0.59) | 2,896 (0.59) | 2,899 (0.59) | 2,900 (0.59) | 2,901 (0.59) | 2,900 (0.59) |
| Asian or Asian British | 9,496 (1.92) | 9,555 (1.94) | 9,559 (1.94) | 9,563 (1.94) | 9,555 (1.94) | 9,563 (1.94) |
| Black or Black British | 7,790 (1.58) | 7,852 (1.59) | 7,841 (1.59) | 7,844 (1.59) | 7,859 (1.59) | 7,867 (1.59) |
| Other | 5,941 (1.20) | 5,970 (1.21) | 5,978 (1.21) | 5,968 (1.21) | 5,972 (1.21) | 5,970 (1.21) |
| Townsend score | -1.31 (3.09) | -1.31 (3.09) | -1.31 (3.09) | -1.31 (3.09) | -1.31 (3.09) | -1.31 (3.09) |
| Smoking (%) |  |  |  |  |  |  |
| Never smoked | 269,099 (54.50) | 270,585 (54.80) | 270,581 (54.80) | 270,555 (54.79) | 270,595 (54.80) | 270,556 (54.79) |
| Previous smoker | 170,287 (34.49) | 171,030 (34.64) | 171,003 (34.63) | 171,089 (34.65) | 171,041 (34.64) | 171,067 (34.64) |
| Current smoker | 51,887 (10.51) | 52,159 (10.56) | 52,190 (10.57) | 52,130 (10.56) | 52,138 (10.56) | 52,151 (10.56) |
| Drinking (%) |  |  |  |  |  |  |
| Daily or almost daily | 100,436 (20.34) | 100,613 (20.38) | 100,609 (20.38) | 100,587 (20.37) | 100,614 (20.38) | 100,608 (20.38) |
| Three or four times a week | 113,986 (23.08) | 114,143 (23.12) | 114,170 (23.12) | 114,177 (23.12) | 114,144 (23.12) | 114,140 (23.12) |
| Once or twice a week | 127,356 (25.79) | 127,603 (25.84) | 127,629 (25.85) | 127,600 (25.84) | 127,611 (25.84) | 127,609 (25.84) |
| One to three times a month | 54,949 (11.13) | 55,053 (11.15) | 55,058 (11.15) | 55,069 (11.15) | 55,062 (11.15) | 55,075 (11.15) |
| Special occasions only | 56,688 (11.48) | 56,874 (11.52) | 56,844 (11.51) | 56,877 (11.52) | 56,870 (11.52) | 56,865 (11.52) |
| Never | 39,273 (7.95) | 39,488 (8.00) | 39,464 (7.99) | 39,464 (7.99) | 39,473 (7.99) | 39,477 (7.99) |
| Physical activity (%) |  |  |  |  |  |  |
| Low | 73,913 (14.97) | 92,741 (18.78) | 92,893 (18.81) | 92,871 (18.81) | 92,780 (18.79) | 92,960 (18.83) |
| Moderate | 200,170 (40.54) | 248,450 (50.32) | 248,398 (50.31) | 248,408 (50.31) | 248,530 (50.33) | 248,324 (50.29) |
| High | 121,942 (24.70) | 152,583 (30.90) | 152,483 (30.88) | 152,495 (30.88) | 152,464 (30.88) | 152,490 (30.88) |
| BMI (kg/m^2^) | 27.42 (4.79) | 27.42 (4.79) | 27.42 (4.79) | 27.42 (4.79) | 27.42 (4.79) | 27.42 (4.79) |
| SBP (mmHg) | 137.78 (18.62) | 137.77 (18.58) | 137.77 (18.58) | 137.77 (18.58) | 137.77 (18.58) | 137.76 (18.58) |
| DBP (mmHg) | 82.23 (10.11) | 82.24 (10.09) | 82.23 (10.09) | 82.23 (10.09) | 82.24 (10.10) | 82.23 (10.09) |

Data were presented as frequency (%) or mean (standard deviation).

Abbreviations: BMI, body mass index; SBP, systolic blood pressure; DBP, diastolic blood pressure.

**Table S5** Likelihood ratio tests of interactions between handgrip strength and age, sex or physical activity

|  | HGS*age  (*P* value) | HGS*sex  (*P* value) | HGS*physical activity (*P* value) |
| --- | --- | --- | --- |
| From no cardiometabolic diseases to CM | 0.4404 | 0.0011 | 0.1545 |
| From type 2 diabetes to CM | 0.6952 | 0.0357 | 0.2170 |
| From CHD to CM | 0.2279 | 0.0583 | 0.1642 |
| From stroke to CM | 0.0202 | 0.1303 | 0.5078 |
| From CM to all-cause mortality | 0.9390 | 0.2903 | 0.0486 |

Abbreviations: HGS, handgrip strength; CHD, coronary heart disease; CM, cardiometabolic multimorbidity.

**Table S6** Associations of handgrip strength with risks of all transitions from baseline to individual cardiometabolic disease, then to subsequent CM and ultimately to death

| **Transitions** | **Number of cases** |  | **Hazard ratios (95% confidence intervals)** | | | | |
| --- | --- | --- | --- | --- | --- | --- | --- |
|  |  |  | **Q4** | **Q3** | **Q2** | **Q1** | **Continuous variable^a^** |
| A (baseline to one cardiometabolic disease) | 47,301/441,868 |  | 1 (reference) | 1.08 (1.05-1.11) | 1.17 (1.14-1.21) | 1.34 (1.30-1.38) | 1.13 (1.12-1.14) |
| B (one cardiometabolic disease to CM) | 4,701/47,301 |  | 1 (reference) | 1.01 (0.91-1.12) | 1.10 (1.00-1.21) | 1.16 (1.05-1.28) | 1.06 (1.03-1.10) |
| C (baseline to death) | 17,337/441,868 |  | 1 (reference) | 1.02 (0.97-1.07) | 1.13 (1.08-1.18) | 1.35 (1.29-1.42) | 1.15 (1.13-1.17) |
| D (one cardiometabolic disease to death) | 6,781/47,301 |  | 1 (reference) | 1.13 (1.03-1.24) | 1.14 (1.04-1.24) | 1.32 (1.21-1.44) | 1.11 (1.08-1.15) |
| E (CM to death) | 1,124/4,701 |  | 1 (reference) | 0.98 (0.78-1.23) | 1.03 (0.83-1.28) | 1.15 (0.94-1.42) | 1.10 (1.03-1.18) |

Models were adjusted for age, sex, socioeconomic status, ethnicity, smoking status, alcohol drinking, physical activity, BMI and hypertension.

^a^ Continuous variable was represented by per standard deviation (SD) decrease of handgrip strength.

Abbreviations: CM, cardiometabolic multimorbidity; Q1, the first quartile; Q2, the second quartile; Q3, the third quartile; Q4, the last quartile.

**Table S7** Sensitivity analyses for the associations of handgrip strength with the risk of cardiometabolic multimorbidity*

|  | Unadjusted model | |  | Adjusted model | |
| --- | --- | --- | --- | --- | --- |
|  | HR (95% CI) | *P* |  | HR (95% CI) | *P* |
| **Participants without any cardiometabolic disease at baseline (CM cases=4,537)** | | | | | |
| Q4 (highest) | 1 (reference) |  |  | 1 (reference) |  |
| Q3 | 1.30 (1.18-1.43) | <0.0001 |  | 1.07 (0.97-1.17) | 0.1843 |
| Q2 | 1.68 (1.54-1.83) | <0.0001 |  | 1.22 (1.11-1.33) | <0.0001 |
| Q1 (lowest) | 2.38 (2.18-2.58) | <0.0001 |  | 1.45 (1.33-1.59) | <0.0001 |
| Continuous variable^a^ | 1.20 (1.17-1.24) | <0.0001 |  | 1.17 (1.13-1.20) | <0.0001 |
| **Participants with type 2 diabetes at baseline (CM cases=3,049)** | | | | | |
| Q4 (highest) | 1 (reference) |  |  | 1 (reference) |  |
| Q3 | 1.04 (0.94-1.16) | 0.4542 |  | 1.00 (0.89-1.11) | 0.9218 |
| Q2 | 1.38 (1.26-1.53) | <0.0001 |  | 1.15 (1.04-1.27) | 0.0062 |
| Q1 (lowest) | 1.69 (1.53-1.88) | <0.0001 |  | 1.33 (1.20-1.48) | <0.0001 |
| Continuous variable | 1.13 (1.09-1.17) | <0.0001 |  | 1.12 (1.08-1.16) | <0.0001 |
| **Participants with stroke at baseline (CM cases=1,082)** | | | | | |
| Q4 (highest) | 1 (reference) |  |  | 1 (reference) |  |
| Q3 | 1.05 (0.89-1.24) | 0.5702 |  | 0.99 (0.83-1.17) | 0.8595 |
| Q2 | 1.22 (1.03-1.45) | 0.0219 |  | 1.07 (0.90-1.28) | 0.4234 |
| Q1 (lowest) | 1.46 (1.23-1.73) | <0.0001 |  | 1.14 (0.96-1.36) | 0.1479 |
| Continuous variable | 1.07 (1.01-1.13) | 0.0166 |  | 1.09 (1.02-1.16) | 0.0095 |
| **Participants with CHD at baseline (CM cases=2,936)** | | | | | |
| Q4 (highest) | 1 (reference) |  |  | 1 (reference) |  |
| Q3 | 1.21 (1.09-1.34) | 0.0003 |  | 1.18 (1.06-1.31) | 0.0021 |
| Q2 | 1.27 (1.15-1.41) | <0.0001 |  | 1.18 (1.06-1.31) | 0.0020 |
| Q1 (lowest) | 1.42 (1.28-1.57) | <0.0001 |  | 1.22 (1.10-1.36) | 0.0003 |
| Continuous variable | 1.07 (1.04-1.11) | <0.0001 |  | 1.08 (1.04-1.12) | <0.0001 |

Adjusted model was adjusted for age, sex, socioeconomic status, ethnicity, smoking status, alcohol drinking, physical activity, BMI and hypertension.

* Sensitivity analyses were conducted by adding a new variable=entry_time+2 (years) as entry time.

^a^ Continuous variable was represented by per standard deviation (SD) decrease of handgrip strength.

Abbreviations: HR, hazard ratio; CI, confidence interval; Q1, the first quartile; Q2, the second quartile; Q3, the third quartile; Q4, the last quartile; CHD, coronary heart disease; CM, cardiometabolic multimorbidity.

**Table S8** Sensitivity analyses for the associations of handgrip strength with the risk of all-cause mortality among patients with cardiometabolic multimorbidity *

|  | Unadjusted model | |  | Adjusted model | |
| --- | --- | --- | --- | --- | --- |
|  | HR (95% CI) | *P* |  | HR (95% CI) | *P* |
| Q4 (highest) | 1 (reference) |  |  | 1 (reference) |  |
| Q3 | 1.34 (1.17-1.53) | <0.0001 |  | 1.22 (1.07-1.40) | 0.0041 |
| Q2 | 1.43 (1.24-1.65) | <0.0001 |  | 1.19 (1.03-1.37) | 0.0205 |
| Q1 (lowest) | 1.82 (1.58-2.10) | <0.0001 |  | 1.51 (1.31-1.75) | <0.0001 |
| Continuous variable^a^ | 1.11 (1.06-1.16) | <0.0001 |  | 1.13 (1.07-1.19) | <0.0001 |

Adjusted model was adjusted for age, sex, socioeconomic status, ethnicity, smoking status, alcohol drinking, physical activity, BMI and hypertension.

* Sensitivity analyses were conducted by adding a new variable=entry_time+2 (years) as the entry time.

^a^ Continuous variable was represented by per standard deviation (SD) decrease of handgrip strength.

Abbreviations: HR, hazard ratio; CI, confidence interval; Q1, the first quartile; Q2, the second quartile; Q3, the third quartile; Q4, the last quartile.


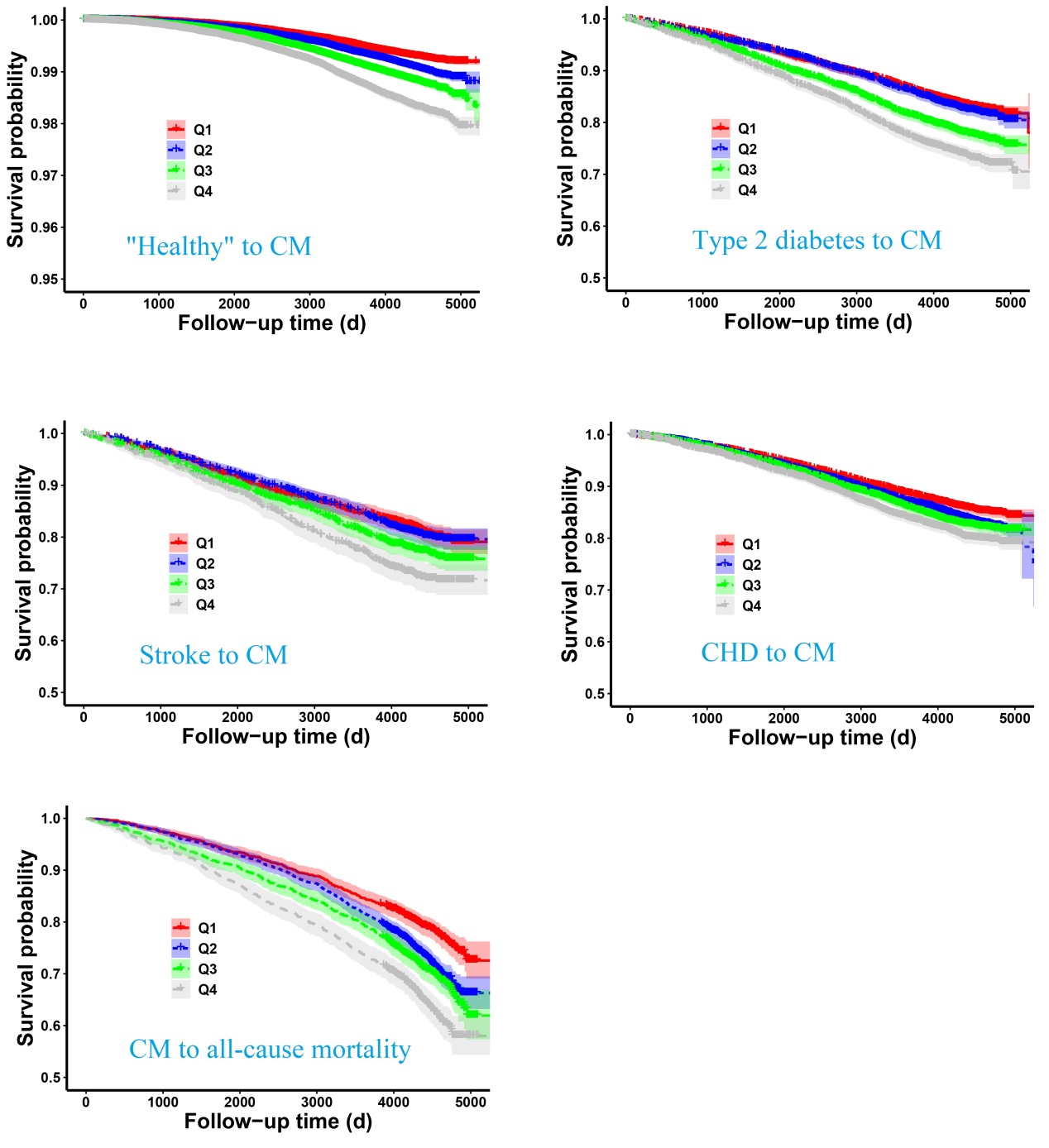


**Fig. S1** Kaplan-Meier curves stratified by HGS quartiles


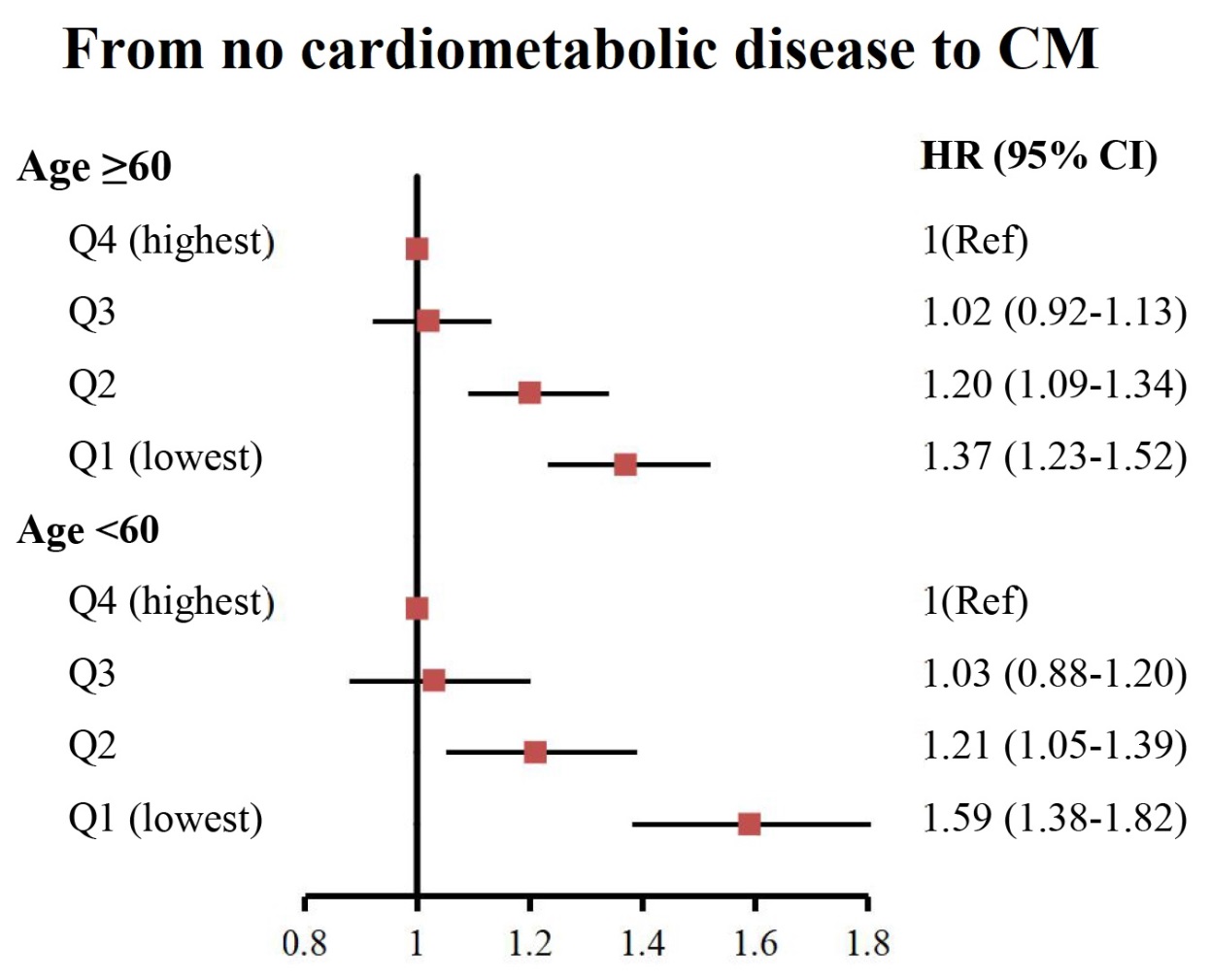


**Fig. S2** Subgroup analyses by age for the association of baseline handgrip strength with the risk of follow-up cardiometabolic multimorbidity among participants free of cardiometabolic disease at baseline

**
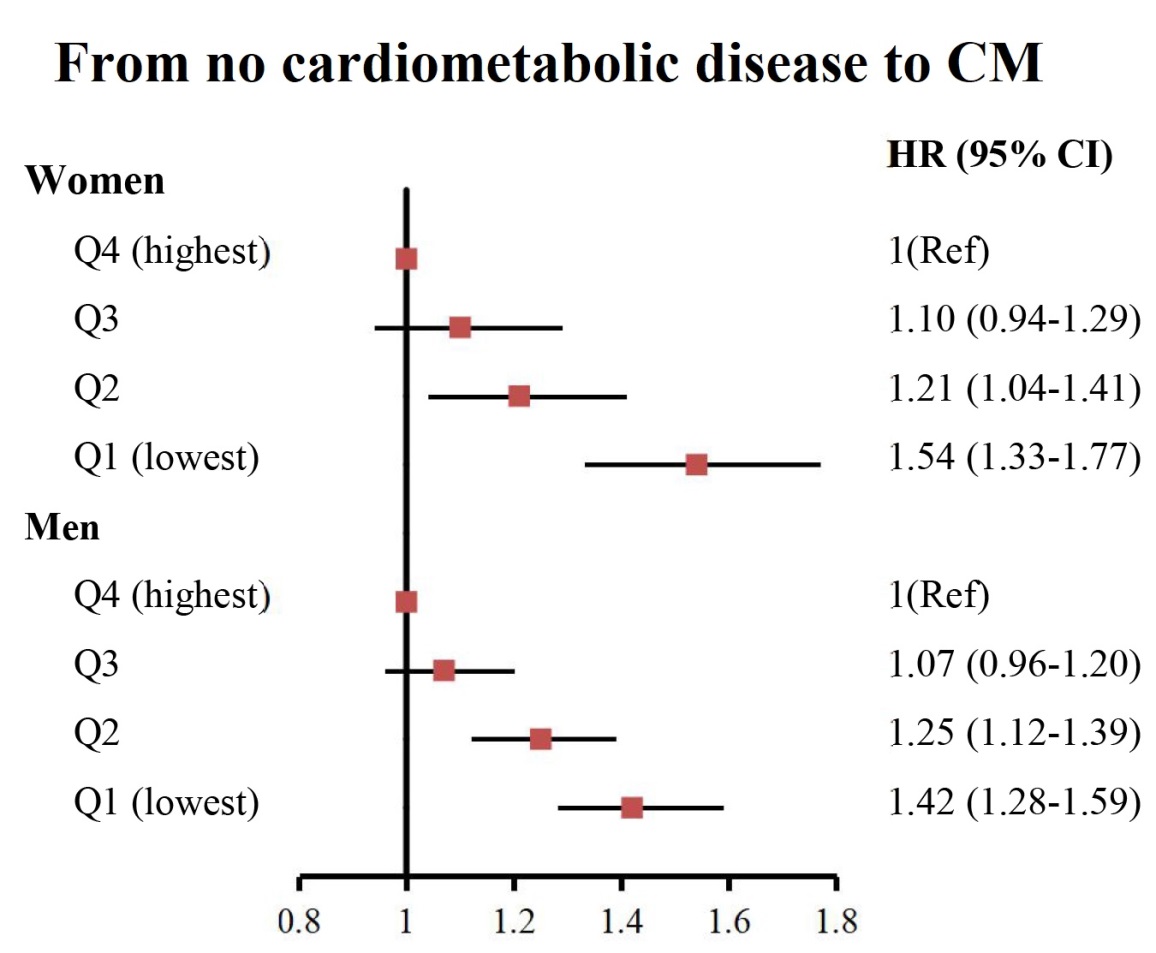
**

**Fig. S3** Subgroup analyses by sex for the association of baseline handgrip strength with the risk of follow-up cardiometabolic multimorbidity among participants free of cardiometabolic disease at baseline


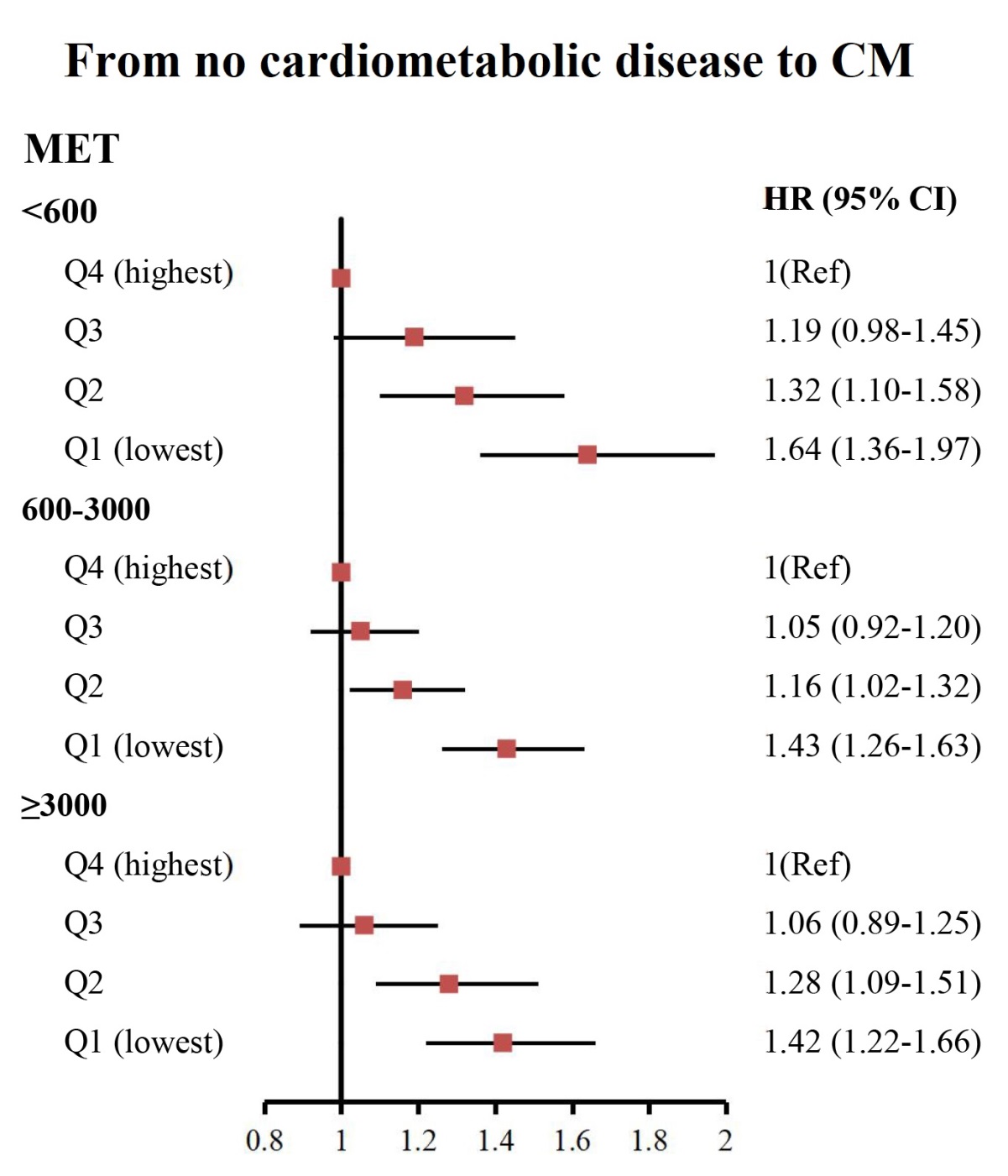


**Fig. S4** Subgroup analyses by physical activity levels for the association of baseline handgrip strength with the risk of follow-up cardiometabolic multimorbidity among participants free of cardiometabolic disease at baseline


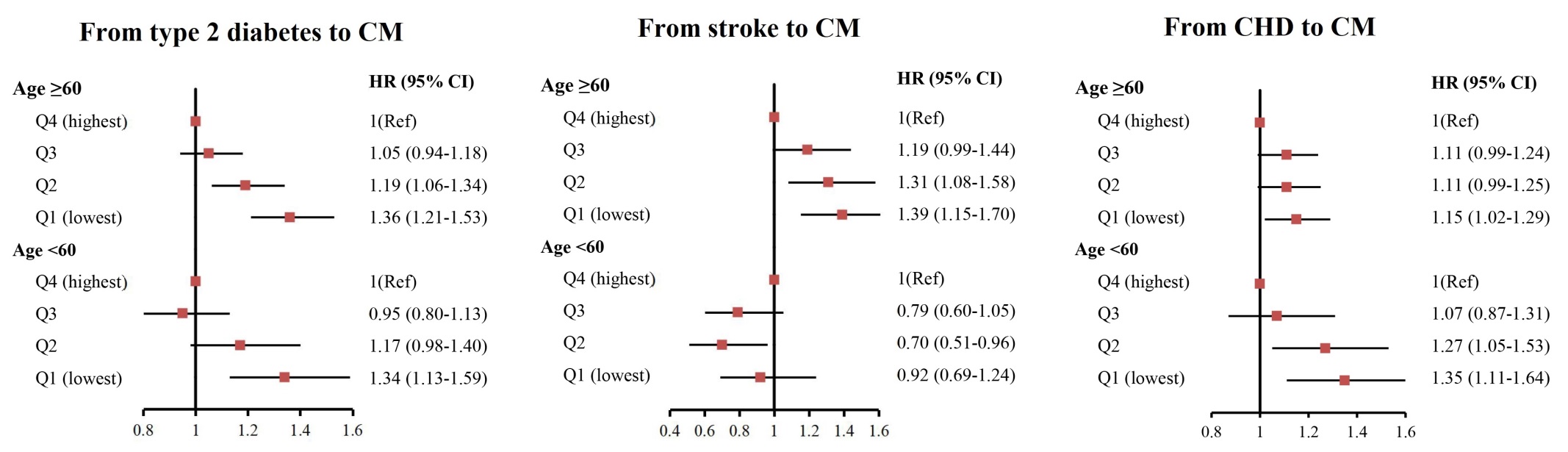


**Fig. S5** Subgroup analyses by age for the association of baseline handgrip strength with the risk of follow-up cardiometabolic multimorbidity among patients with one cardiometabolic disease at baseline

**
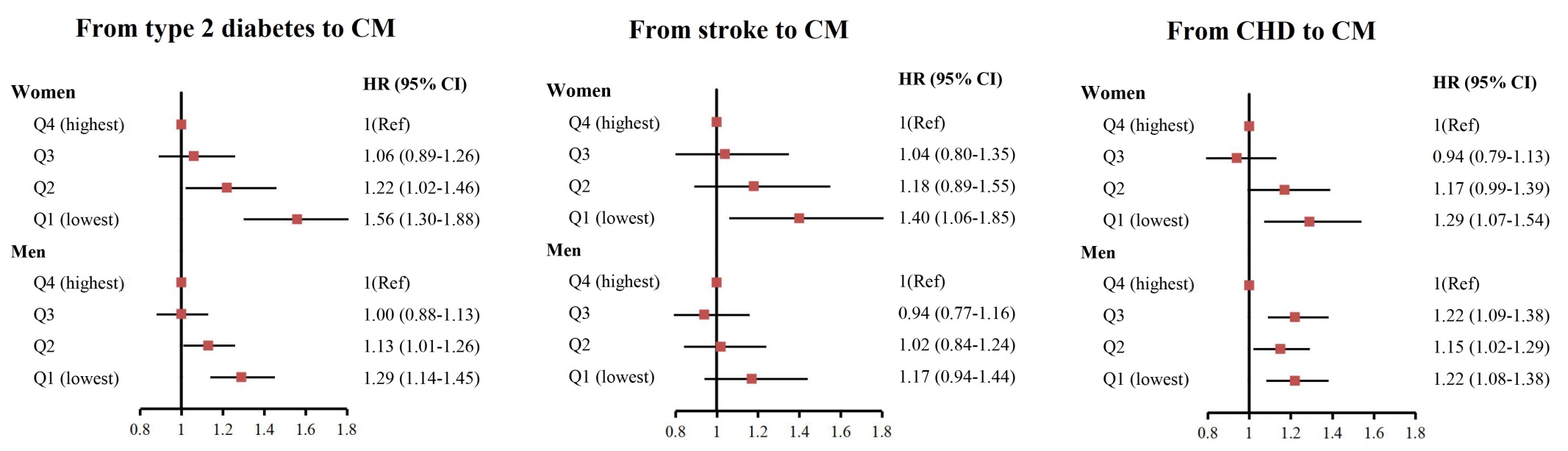
**

**Fig. S6** Subgroup analyses by sex for the association of baseline handgrip strength with the risk of follow-up cardiometabolic multimorbidity among patients with one cardiometabolic disease at baseline


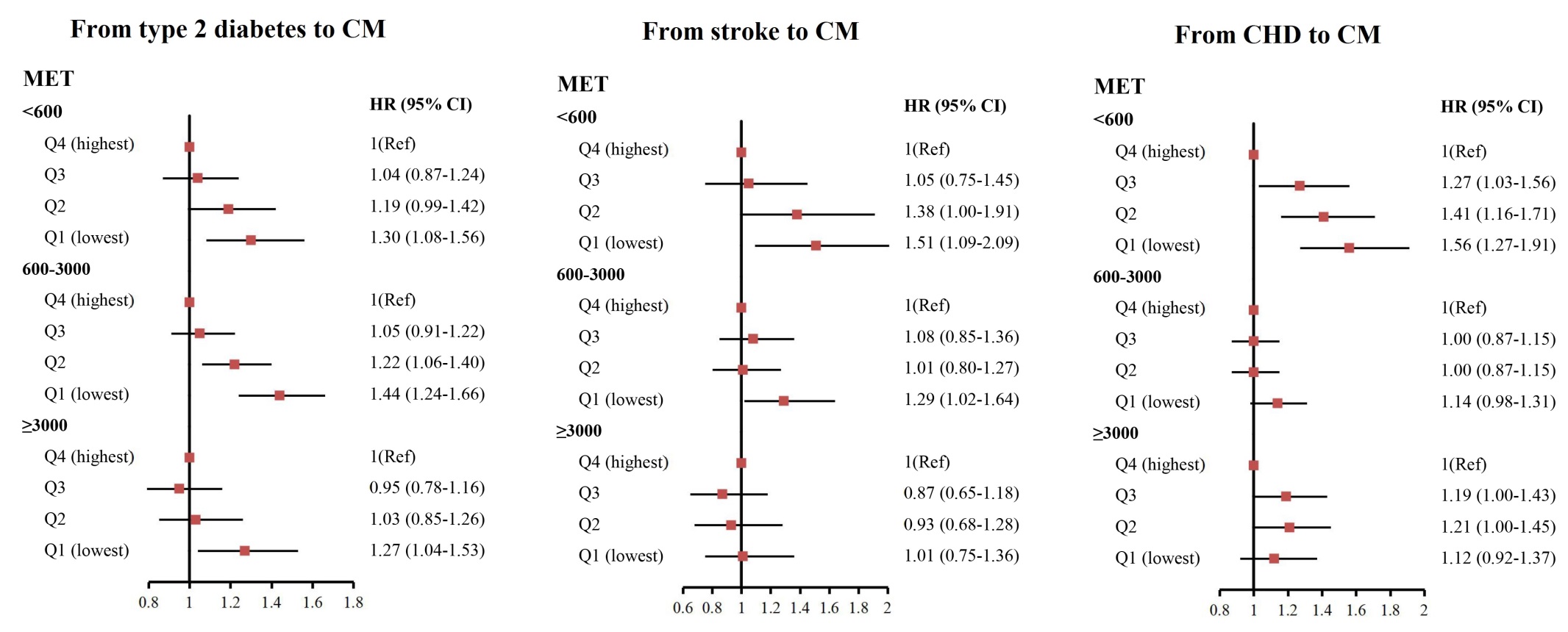


**Fig. S7** Subgroup analyses by physical activity levels for the association of baseline handgrip strength with the risk of follow-up cardiometabolic multimorbidity among patients with one cardiometabolic disease at baseline

**
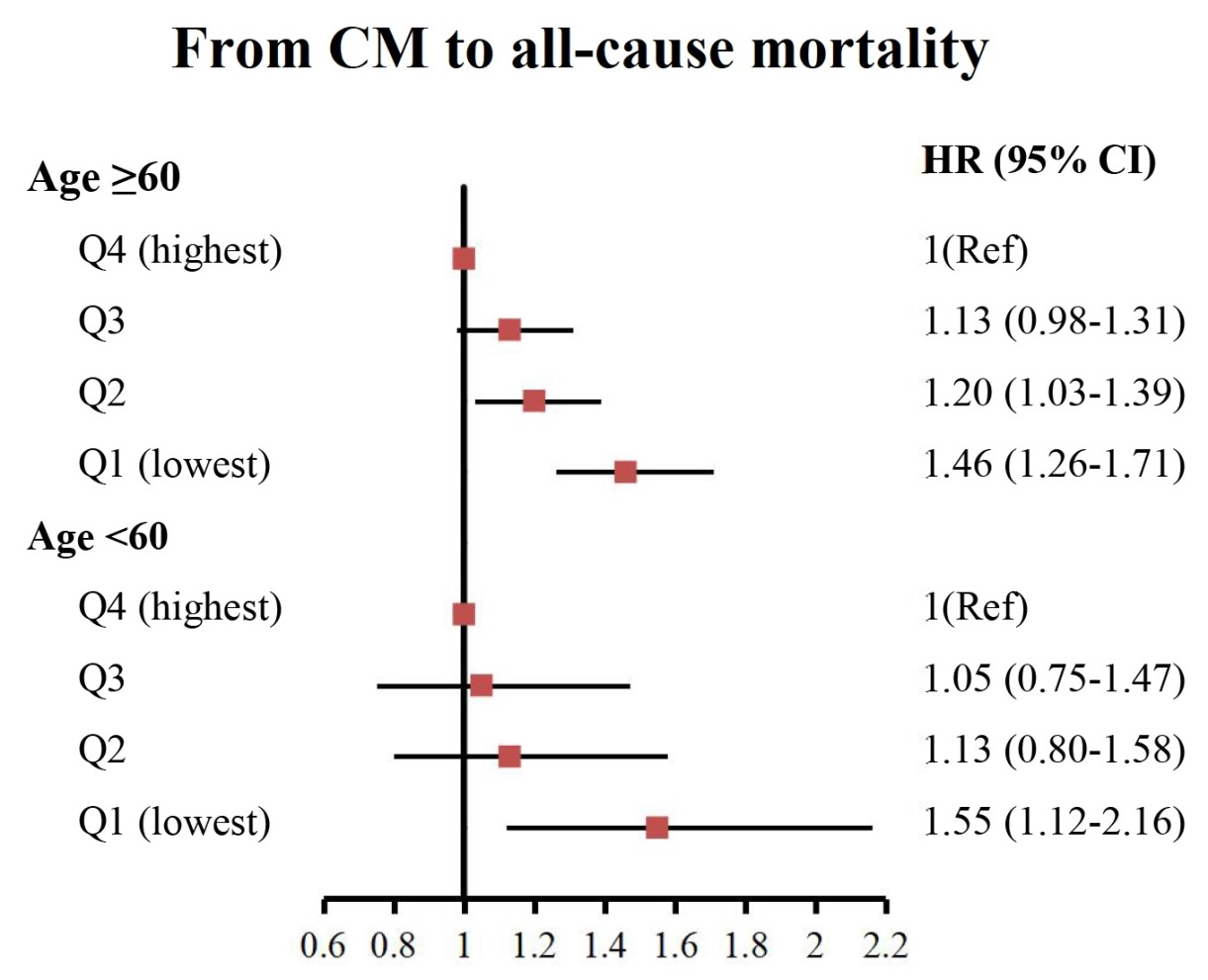
**

**Fig. S8** Subgroup analyses by age for the association of baseline handgrip strength with the risk of follow-up all-cause mortality among patients with cardiometabolic multimorbidity at baseline


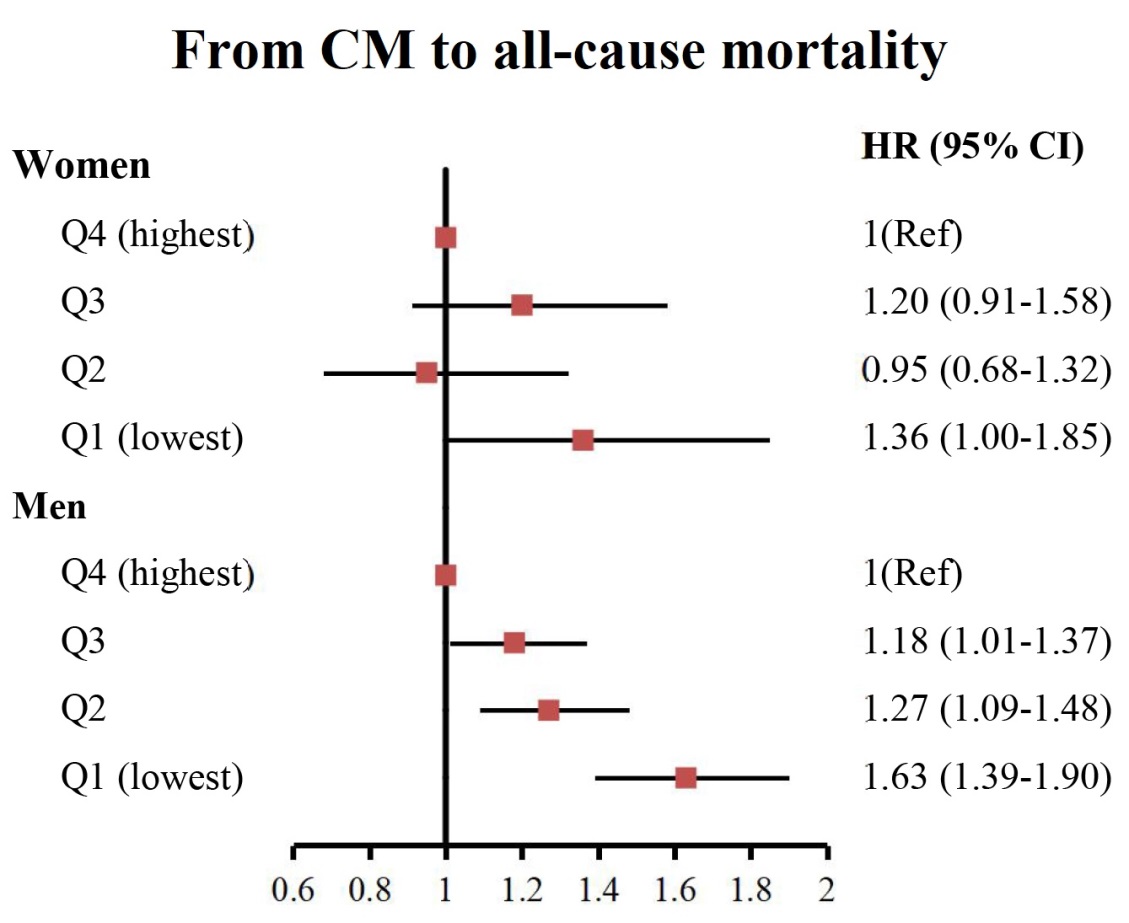


**Fig. S9** Subgroup analyses by sex for the association of baseline handgrip strength with the risk of follow-up all-cause mortality among patients with cardiometabolic multimorbidity at baseline


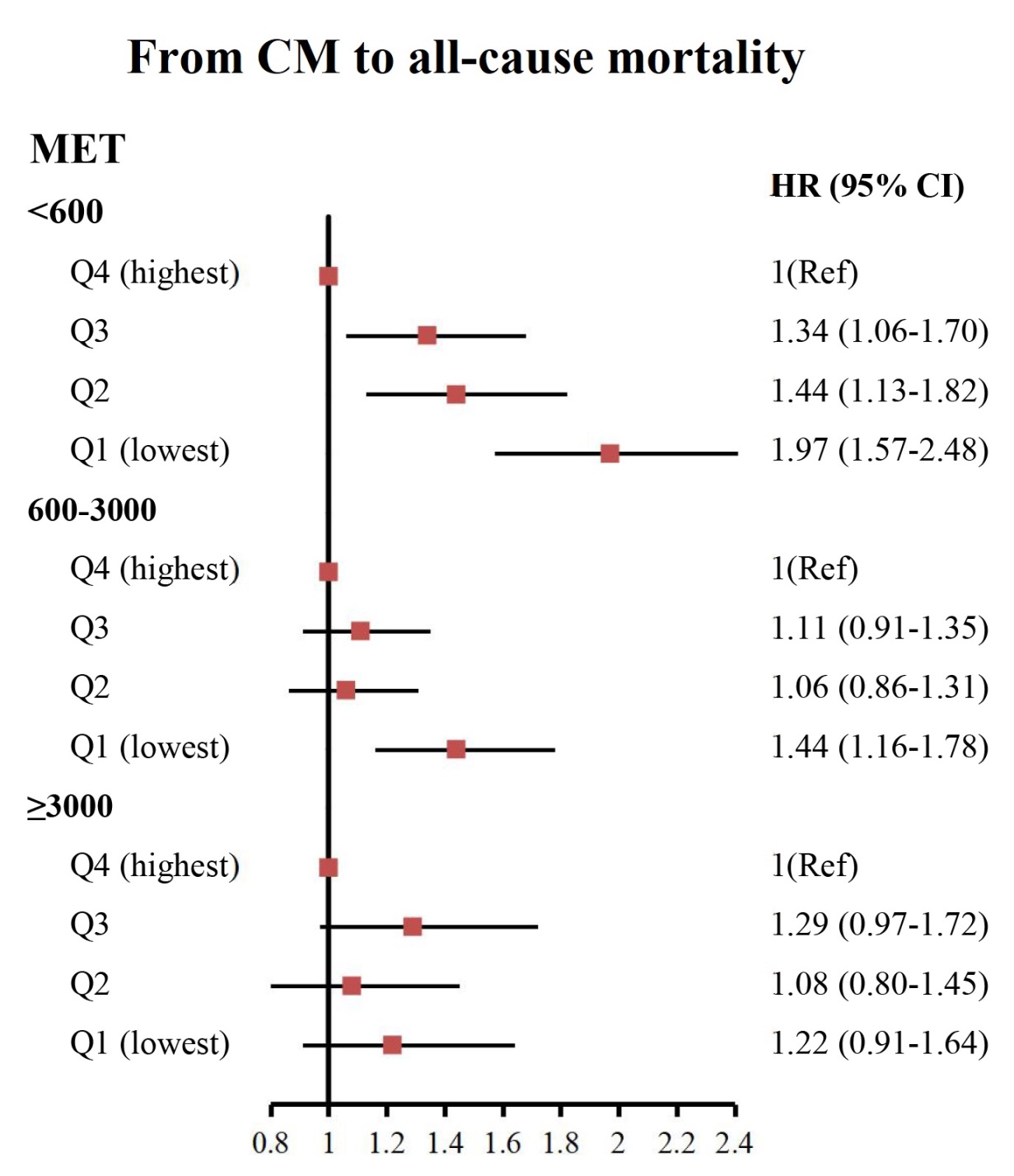


**Fig. S10** Subgroup analyses by physical activity levels for the association of baseline handgrip strength with the risk of follow-up all-cause mortality among patients with cardiometabolic multimorbidity at baseline
